# Intolerance of uncertainty and vaccine hesitancy of health care workers in response to covid 19 vaccination

**DOI:** 10.1192/j.eurpsy.2023.1711

**Published:** 2023-07-19

**Authors:** S. Dhakouani, R. Lansari, N. hamrouni, A. Larnaout, W. melki

**Affiliations:** 1 psychiatry, Razi hospital, tunis, Tunisia

## Abstract

**Introduction:**

The covid 19 pandemic was a period of uncertainty. This uncertainty was sustained even after the advent of the vaccine against covid 19. Several concerns have emerged related to the vaccine and health care workers were at the centre of this uncertainty.

**Objectives:**

Study intolerance of uncertainty and vaccine hesitancy among health care workers in relation to covid 19 vaccination

**Methods:**

This is a descriptive study conducted by a questionnaire posted on social networks using Google Forms targeting groups of health professionals before the launch of the vaccination campaign in Tunisia from January 16, 2021 to March 6, 2021.

We collected sociodemographic data and the attitudes of health care workers about COVID 19 vaccination. We used Intolerance of Uncertainty Scale short form (IUS 12) to evaluate the intolerance of uncertainty related to COVID 19 vaccine

**Results:**

Our study included 168 health care workers represented mainly by medical personnel (81% of the respondents). The average age was 34 ±10 years and sex ratio was 0.22.

Sixty percent (60%) of population were hesitant in front of the COVID 19 vaccine.

This hesitancy was explained in 90% of cases by the insecurity of the new COVID19 vaccine. Thirty-seven percent (37%) doubted the efficacy of these vaccines and 22% trivialized COVID19 by expressing the worthlessness of preventing this disease.

The mean score for intolerance of uncertainty was 26.57 ± 9.68 and a median of 26. The minimum score was 10 and the maximum score was 50. We found a significant association between intolerance of uncertainty and vaccine hesitancy (p=0.034). Subjects with higher uncertainty intolerance scores were less hesitant in front of COVID19 vaccination.

**Conclusions:**

Intolerance of uncertainty is a consideration when introducing a new covid 19 vaccine to health care workers and in a pandemic context

**Disclosure of Interest:**

None Declared

